# HIV-1 integrase inhibitor resistance in viraemic-treated individuals and baseline capsid diversity relevant to future lenacapavir therapy

**DOI:** 10.1093/jac/dkag300

**Published:** 2026-09-08

**Authors:** Adam Abdullahi, Sophia Osawe, Haruna Wisso, Martin Edun, Grace Adebisi, Judith Arachie, Chinedu Ude, James Onyemata, Suleiman Yusuf Alhaji, Yusuf Bara Jibrin, Alash’le Abimiku, Steven A Kemp, Ravindra K Gupta

**Affiliations:** Department of Medicine, University of Cambridge, Cambridge Institute of Therapeutic Immunology and Infectious Disease (CITIID), Cambridge, UK; International Research Centre of Excellence, Institute of Human Virology, Abuja, Nigeria; Department of Immunology and Infectious Diseases, Harvard T.H. Chan School of Public Health, Boston, MA, USA; Hong Kong Jockey Club Global Health Institute, Hong Kong, SAR, China; International Research Centre of Excellence, Institute of Human Virology, Abuja, Nigeria; International Research Centre of Excellence, Institute of Human Virology, Abuja, Nigeria; International Research Centre of Excellence, Institute of Human Virology, Abuja, Nigeria; International Research Centre of Excellence, Institute of Human Virology, Abuja, Nigeria; International Research Centre of Excellence, Institute of Human Virology, Abuja, Nigeria; International Research Centre of Excellence, Institute of Human Virology, Abuja, Nigeria; International Research Centre of Excellence, Institute of Human Virology, Abuja, Nigeria; Molecular Genetics & Infectious Diseases Research Laboratory, Abubakar Tafawa Balewa University Teaching Hospital, Bauchi 740101, Nigeria; Department of Medicine, Abubakar Tafawa Balewa University Teaching Hospital, Bauchi 740272, Nigeria; International Research Centre of Excellence, Institute of Human Virology, Abuja, Nigeria; Department of Medicine, University of Cambridge, Cambridge Institute of Therapeutic Immunology and Infectious Disease (CITIID), Cambridge, UK; Department of Medicine, University of Cambridge, Cambridge Institute of Therapeutic Immunology and Infectious Disease (CITIID), Cambridge, UK; Hong Kong Jockey Club Global Health Institute, Hong Kong, SAR, China; Africa Health Research Institute, Durban, South Africa

## Abstract

**Background:**

Dolutegravir (DTG)-based antiretroviral therapy has been widely adopted across sub-Saharan Africa, yet resistance pathways in non-B HIV-1 subtypes remain poorly defined. As lenacapavir (LEN), a long-acting capsid inhibitor, is introduced for prevention and treatment, characterizing baseline capsid variation alongside emerging integrase resistance is increasingly important. Whether DTG resistance pathways coexist with known capsid resistance mutations in West African epidemics remains poorly described.

**Methods:**

We performed deep sequencing of 87 HIV-1 samples from adults (*n* = 47) and paediatric participants (*n* = 40) failing DTG-based antiretroviral therapy in Nigeria. Resistance mutations were interpreted using Stanford HIVdb and IAS-USA criteria, with resistance estimates based on gene- or drug-panel-specific denominators. Low-frequency variants were assessed at 2%, 5%, 10% and 20% read-frequency thresholds. Capsid mutations associated with LEN resistance were characterized, structurally mapped and compared to global sequence data. HIV-1 subtype and recombinant forms were assigned using COMET HIV-1 v2.4.

**Results:**

At consensus, predicted resistance to ≥1 integrase strand transfer inhibitor (INSTI) was detected in 13/50 integrase-genotypable participants (26.0%; 95% CI 15.9%–39.6%), including 5/50 with a major INSTI resistance mutation. Reduced predicted susceptibility specifically to DTG was present in 6/50 (12.0%; 95% CI 5.6%–23.8%). G118R was detected in 4/50 participants at consensus, while Q148H/K/R and N155H were absent. In exploratory deep-sequencing analysis, G118R was detected in 8/47 participants with sufficient codon-level coverage at the ≥20% read-frequency threshold. No known primary LEN resistance mutation was detected among 33 participants with complete coverage of all six primary resistance positions (0/33; 95% CI 0%–10.4%); T107A, a polymorphic capsid substitution of uncertain effect on LEN susceptibility, was detected in in 3/33 individuals where sequence could be evaluated.

**Conclusions:**

These findings demonstrate DTG resistance in both adult and paediatric participants with virological failure with G118R emerging as a prominent resistance pathway. No known primary LEN resistance mutation was detected in the assessable capsid sequences, although phenotypic susceptibility and clinical outcomes were not evaluated. These findings support continued surveillance of integrase and capsid resistance as long-acting antiretroviral strategies expand.

## Introduction

Since 2018, the World Health Organization has recommended integrase strand transfer inhibitor (INSTI)-based regimens, particularly dolutegravir (DTG), as the preferred first-line antiretroviral therapy (ART).^1–3^ This rapid programmatic transition, in high-burden and low-resource countries, was driven by rising efavirenz-based ART resistance and DTG’s favourable efficacy and tolerability.^4–8^ However, widespread DTG use inevitably exerts selective pressure on HIV-1 integrase, making it crucial to understand which resistance pathways emerge in real-world, non-B subtype epidemics.

INSTI resistance arises through characteristic mutational pathways, typically involving primary substitutions, such as G118R, Q148H/K/R and N155H, together with accessory mutations that modulate resistance and viral fitness.^9–15^ Most pathway data come from subtype B epidemics in high-resource settings, where Q148- and N155-centred routes predominate. Whether the relative use of these pathways differs in non-B integrase backgrounds remains uncertain. As DTG coverage expands across West and Central Africa, where CRF02_AG, subtype G and diverse recombinant forms dominate, context-specific data on INSTI pathways selected during routine DTG use are needed.^14–16^

In parallel, lenacapavir (LEN), a first in class long-acting capsid inhibitor, has shown high efficacy for HIV prevention and is approved for multidrug-resistant HIV-1 infection.^17–19^ Recent structural work shows that integrase anchors viral RNA within the capsid core, while the capsid regulates nuclear trafficking, uncoating and chromatin targeting of the pre-integration complex.^20^ This links the two proteins during early infection, although it does not imply cross-resistance between integrase and capsid inhibitors. Systematic rollout of LEN has begun across sub-Saharan Africa,^21,22^ supported by global access agreements aimed at expanding equitable access in high-burden settings.^23^ In context, LEN resistance in clinical and preclinical studies associates with capsid substitutions (M66I, Q67H, K70H/N, N74D),^24,25^ yet baseline prevalence of these mutations in African non-B epidemics remains poorly characterized. A previous study found no enrichment of GS-6207 resistance-associated capsid substitutions in protease inhibitor-experienced patients.^26^ For programmes deploying LEN and long-acting cabotegravir plus rilpivirine (CAB + RPV) alongside widespread DTG use, a key question is whether integrase resistance observed during DTG-based ART, including G118R, coexists with known capsid resistance mutations and how often low-frequency variants are present.

Nigeria provides an ideal setting to address these questions. It has a large, predominantly non-B HIV-1 epidemic, adopted DTG-based first-line ART in 2018 with rapid uptake and carries a substantial paediatric HIV burden with incomplete prevention of mother-to-child transmission (PMTCT) coverage.^1–3,27,28^ Paediatric HIV presents distinct challenges to optimal ART outcomes: maternal ART exposure through PMTCT, distinctive pharmacokinetics relative to adults, delayed diagnosis and historical reliance on non-nucleoside reverse transcriptase inhibitor (NNRTI)-based paediatric formulations may shape viral populations differently from adults.^29–32^ Beyond biology, children/adolescents face higher rates of viral coinfections, malnutrition and socio-behavioural barriers, including stigma,^33^ all of which impede virological control. Here, we deep sequenced 87 HIV-1 samples from adults (*n* = 47) and paediatric participants (*n* = 40) failing DTG-based ART in North-Central Nigeria. We applied dual resistance scoring (Stanford HIVdb v10.1^34^ and IAS-USA 2025 catalogues^35^), assigned subtype and recombinant forms using COMET HIV-1 v2.4, profiled INSTI and non-INSTI resistance across all classes, characterized capsid variation relevant to LEN resistance through mutation profiling and structural mapping (PDB 6V2F), compared findings with global capsid diversity (LANL, *n* = 2847) and assessed low-frequency variants at 2%, 5%, 10% and 20% read-frequency thresholds. Because genome coverage was fragmented and many COMET results were complex or unassigned, subtype comparisons were descriptive.

## Methods

### Study cohort and sample collection

This was a cross-sectional study of HIV-1–infected individuals failing dolutegravir-based antiretroviral therapy (DTG-ART). Plasma samples were obtained from two Nigerian cohorts: adults (*n* = 47) and paediatric participants (*n* = 40), totalling 87 participants sampled through July 2024. Adults were recruited from Asokoro District Hospital and Maitama District Hospital in Abuja, Nigeria. Inclusion criteria were (i) age ≥18 years; (ii) documented virological failure (two consecutive HIV-1 RNA > 1000 copies/mL despite adherence support) on DTG-based ART for ≥6 months; (iii) attendance at routine clinic visits; and (iv) ability to provide informed written consent. Paediatric participants were recruited from the Plateau State Human Virology Research Centre. Inclusion criteria were (i) age 0–17 years; (ii) HIV-1 infection confirmed using the national HIV testing algorithm (iii) receipt of DTG-based ART for ≥6 months; (iv) virological failure as defined above; and (v) parental or guardian consent. Demographic data, where available, were extracted from clinical records and recorded in electronic case report forms. Written informed consent was obtained from all adult participants; parental or guardian consent was obtained for paediatric participants, with child assent where appropriate. The study was approved by FCT Research Ethics Committee with approval number FHREC/2023/01/136/25-07-23 (adult cohort) and IHVN Institutional Review Board with approval IHVN-HREC/240502/App and NHREC/09/23/2010b (paediatric cohort).

### Nucleic acid extraction and sequencing

Viral RNA was extracted from plasma samples using Qiagen kits. Full-length HIV-1 genomes were amplified by RT-PCR and sequenced on Illumina platforms (paired-end 2 × 150 bp or 2 × 250 bp). Libraries were prepared using VSP2 kit and multiplexed for sequencing.

### Bioinformatic analysis

All analyses were performed using a Snakemake (v8.25.5) workflow^36^ with conda environment management. Raw reads were quality filtered using fastp v0.23.4^37^ (minimum Phred Q15, minimum length 50 bp, adapter autodetection, deduplication enabled). Reads were mapped to the HXB2 reference genome (GenBank K03455.1) using minimap2 v2.30.^38^ Consensus sequences were generated using ivar v1.4.3^39^ with minimum depth 10×, minimum base quality Q15 and 50% majority-rule consensus calling. Cohort-level quality assessment used MultiQC v1.25.^40^

### Low-frequency variant calling

Low-frequency variants were called using iVar variants with minimum base quality Q15 and reporting thresholds of 2%, 5%, 10% and 20%. At Drug resistance mutation (DRM) sites, amino acid evidence was recounted directly from the BAM at the codon level. Unmapped, secondary, supplementary, quality-control-failed and duplicate reads were excluded, as were reads with mapping quality <20; all three codon bases required Q15 and an unambiguous alignment. A call required at least two alternate codon-supporting read alignments, including at least one from each strand; paired reads were not collapsed to unique molecules, and no formal strand-bias *P* value was applied. The four thresholds required minimum codon depths of 100, 40, 20 and 10 reads, respectively, for two-read detectability. At each threshold, callability was calculated separately for each drug-specific resistance panel. Predictions with no resistance evidence and <30% callable positions were recorded as not assessed; detected resistance evidence was retained. Across 7482 DRM site-participant pairs, median minimum codon depth was 321 (IQR 0–1391); 4,592, 4,876, 5002 and 5081 pairs reached ≥100×, ≥40×, ≥20× and ≥10×, respectively. Negative controls, an empirical sequencing/PCR error estimate, technical replicates, unique molecular identifiers and replicate-concordance estimates were not available, so the low-frequency results were treated as exploratory.

### Drug resistance mutation scoring

Consensus-level resistance was scored using two parallel approaches. First, Stanford HIVdb algorithm v10.1^34^ was applied via sierra-local v0.4.4, a standalone offline implementation scoring concatenated pol sequences (PR + RT + IN) against all approved antiretrovirals. Second, curated YAML-formatted resistance catalogues aligned with the IAS-USA 2025 drug resistance mutations list^35^ were applied using custom Python scripts. Separate catalogues were maintained for nucleoside reverse transcriptase inhibitor (NRTI) (63 mutations), NNRTI (73), PI (79), INSTI (19 IAS-USA 2025 citations including CAB-specific mutations), LEN/capsid (10 IAS-USA 2025 citations), enfuvirtide (gp41) and fostemsavir (gp120). Gene regions were extracted using HXB2 coordinates (PR, 2253–2549; RT, 2550–3869; IN, 4230–5096; CA, 1186–1879), translated and scored against catalogue rules.

Low-frequency resistance-associated mutations were scored separately at the 2%, 5%, 10% and 20% read-frequency thresholds using the custom catalogues. Only variants at DRM positions with sufficient coverage depth were reported. Variants at ≥20% were described as threshold positive rather than minority variants, and threshold-specific scores were not treated as validated clinical susceptibility classifications.

### APOBEC hypermutation screening

Consensus sequences were screened for G→A hypermutation using HYPERMUT 2.0^41^ with Fisher’s exact test (*P* < 0.05) comparing G→A mutations in APOBEC-favoured contexts (GRD) versus non-APOBEC contexts. Hypermutated sequences were flagged to prevent false-positive DRM calls.^42^ No samples (0/87) were flagged.

### HIV-1 subtyping

Subtype assignment was performed using COMET HIV-1 v2.4, accessed 30 July 2026.^43^ We retained the reported subtype or recombinant call and bootstrap support where available. For subtype summaries, sequences were required to contain at least one contiguous 100-nt segment of unambiguous sequence. Six records did not meet this requirement and were treated as not assessable. Exact participant-level results are provided in Supplementary Data S1 (available as Supplementary data at *JAC* Online).

### Phylogenetic analysis

Consensus sequences were aligned against the HXB2 reference and a curated panel of 54 subtype reference sequences using MAFFT v7.525.^44^ Given the fragmented nature of the consensus assemblies (median genome coverage 10.8%), a SNP-based approach was adopted: variant sites present in at least 10 sequences were extracted (6894 SNP positions), and sequences with >90% missing data at these sites were excluded, yielding 125 sequences (87 cohort samples plus 38 references). A maximum-likelihood tree was constructed using IQ-TREE 3 v3.0.1^45^ under the GTR + G4 substitution model with 1000 ultrafast bootstrap replicates. The tree was midpoint rooted.

### Surveillance drug resistance mutation analysis

Surveillance drug resistance mutations (SDRMs) were assessed using the WHO 2009 list for NRTI, NNRTI and PI classes and the Tzou *et al.* (2020) candidate INSTI surveillance mutation list.^13^ Overall SDRM detection prevalence was calculated across all 87 participants; class-specific prevalence used the relevant gene-genotypable denominator. Because all participants were treatment experienced, detected SDRMs were not classified as transmitted resistance.

### Statistical analysis

Cohort comparisons used Fisher’s exact test for categorical variables and Mann–Whitney *U*-test for continuous variables, with Benjamini–Hochberg false discovery rate correction (*q* < 0.05). Wilson score intervals were computed for all proportions. All analyses were performed in Python v3.10 and R v4.4.2.^46^

### Structural analysis

LEN resistance mutations were mapped onto the HIV-1 CA hexamer crystal structure (PDB 6V2F) using PyMOL. Binding-pocket residues were identified based on published LEN–capsid interaction data. Global capsid diversity at LEN resistance positions was assessed from 2847 LANL sequences stratified by subtype. The structural and comparative analyses were used for context and not as phenotypic susceptibility assays.

## Supplementary Material

dkag300_Supplementary_Data

## Figures and Tables

**Figure 1.**
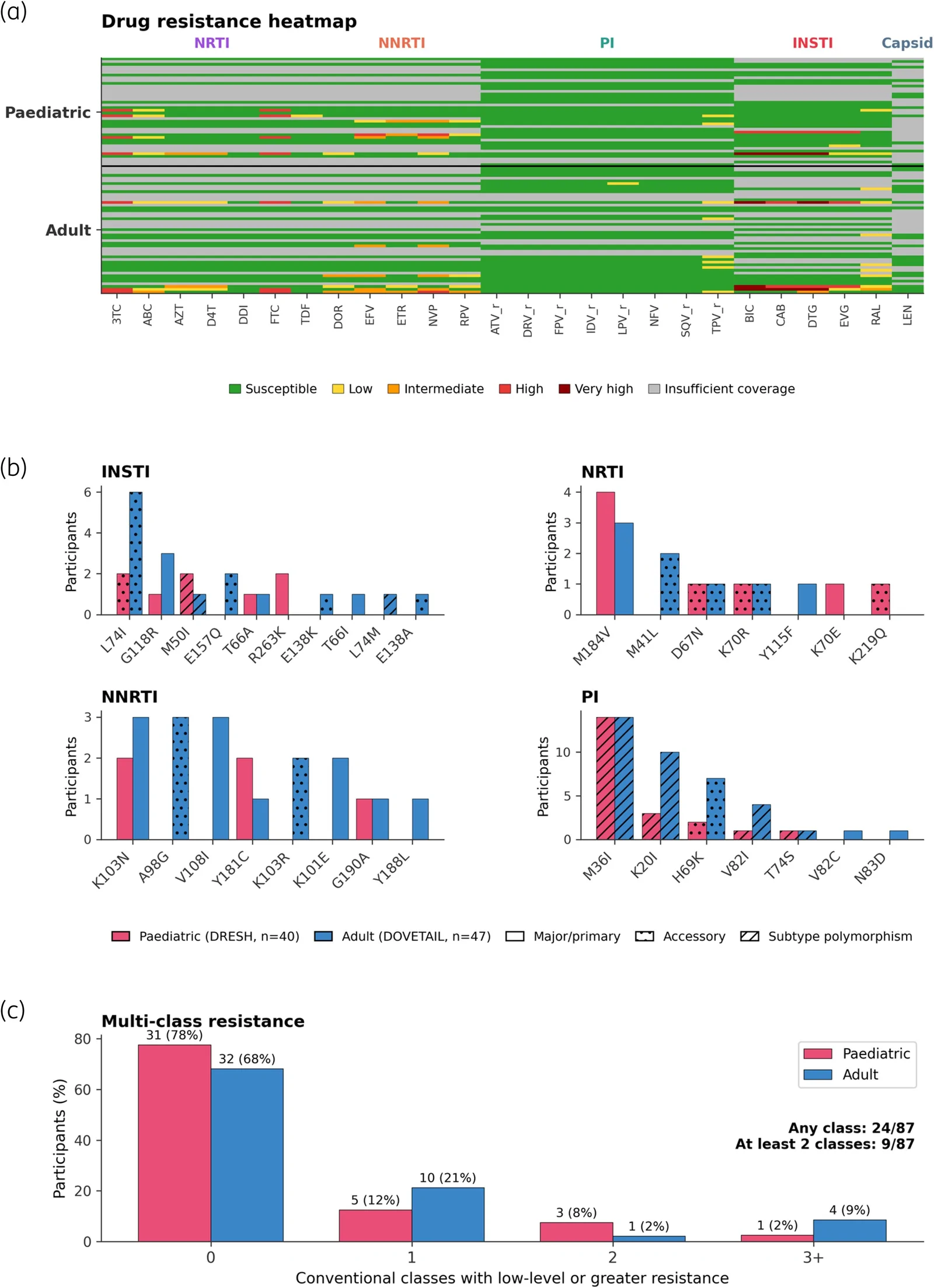
Drug resistance profiles and multi-class resistance in paediatric and adult cohorts. Across the four conventional classes, 24/87 participants had at least one consensus resistance prediction and 9/87 had resistance in two or more classes. (a) Drug resistance heatmap showing predicted susceptibility across antiretroviral drug classes. Paediatric participants are shown above and adults below. Grey denotes insufficient sequence coverage. (b) Prevalence of resistance-associated mutations by class, with primary mutations separated from subtype-associated polymorphisms. (c) Distribution of multi-class resistance by cohort.

**Table 1.** Characteristics of the study population and sequence coverage (*n* = 87)

Characteristic	Total (*n* = 87)	Adults (*n* = 47)	Paediatric participants (*n* = 40)
Female, *n*/*N* (%)	42/74 (56.8)	23/38 (60.5)	19/36 (52.8)
Age (years), median (IQR)	—	41 (36–48)	14 (10.5–17.25)
Time since HIV diagnosis (years), median (IQR)	—	12.2 (9.1–16.0)	4.0 (1.4–7.9)
Plasma HIV-1 RNA at failure (log_10_ copies/mL), median (IQR)	—	4.1 (3.0–5.2)	4.6 (3.4–5.1)
Time on DTG (years), median (IQR)	—	5.0 (4.0–5.5)	2.9 (2.3–4.0)
Prior ART exposure, *n*/*N* (%)			
NNRTI-based regimen	44/73 (60.3)	31/37 (83.8)	13/36 (36.1)
LPV/r-based regimen	20/73 (27.4)	0/37 (0)	20/36 (55.6)
Gene/region genotypable, *n*/*N* (%)			
Protease	65/87 (74.7)	34/47 (72.3)	31/40 (77.5)
Reverse transcriptase	41/87 (47.1)	23/47 (48.9)	18/40 (45.0)
Integrase	50/87 (57.5)	24/47 (51.1)	26/40 (65.0)
Complete primary LEN-site panel	33/87 (37.9)	22/47 (46.8)	11/40 (27.5)
HIV-1 subtype/recombinant assignment, *n*/*N* (%)			
G	18/87 (20.7)	10/47 (21.3)	8/40 (20.0)
CRF02_AG	11/87 (12.6)	6/47 (12.8)	5/40 (12.5)
A1	1/87 (1.1)	1/47 (2.1)	0/40 (0)
Other assigned/check call	7/87 (8.0)	4/47 (8.5)	3/40 (7.5)
Unassigned recombinant profile	44/87 (50.6)	21/47 (44.7)	23/40 (57.5)
Not assessable	6/87 (6.9)	5/47 (10.6)	1/40 (2.5)
DTG suboptimal susceptibility among IN-genotypable, *n*/*N* (%)	6/50 (12.0)	4/24 (16.7)	2/26 (7.7)
Known primary LEN resistance mutation among panel-complete, *n*/*N* (%)	0/33 (0)	0/22 (0)	0/11 (0)

Data are *n*/*N* (%) or median (IQR). Denominators vary according to availability of clinical data and sequence coverage. HIV-1 subtype/recombinant assignments were generated using COMET HIV-1 v2.4; sequences without a contiguous ≥ 100-nt segment of unambiguous sequence were classified as not assessable.

ART, antiretroviral therapy; DTG, dolutegravir; IN, integrase; LEN, lenacapavir; LPV/r, ritonavir-boosted lopinavir; NNRTI, non-nucleoside reverse transcriptase inhibitor; NR, not recorded.

**Figure 2.**
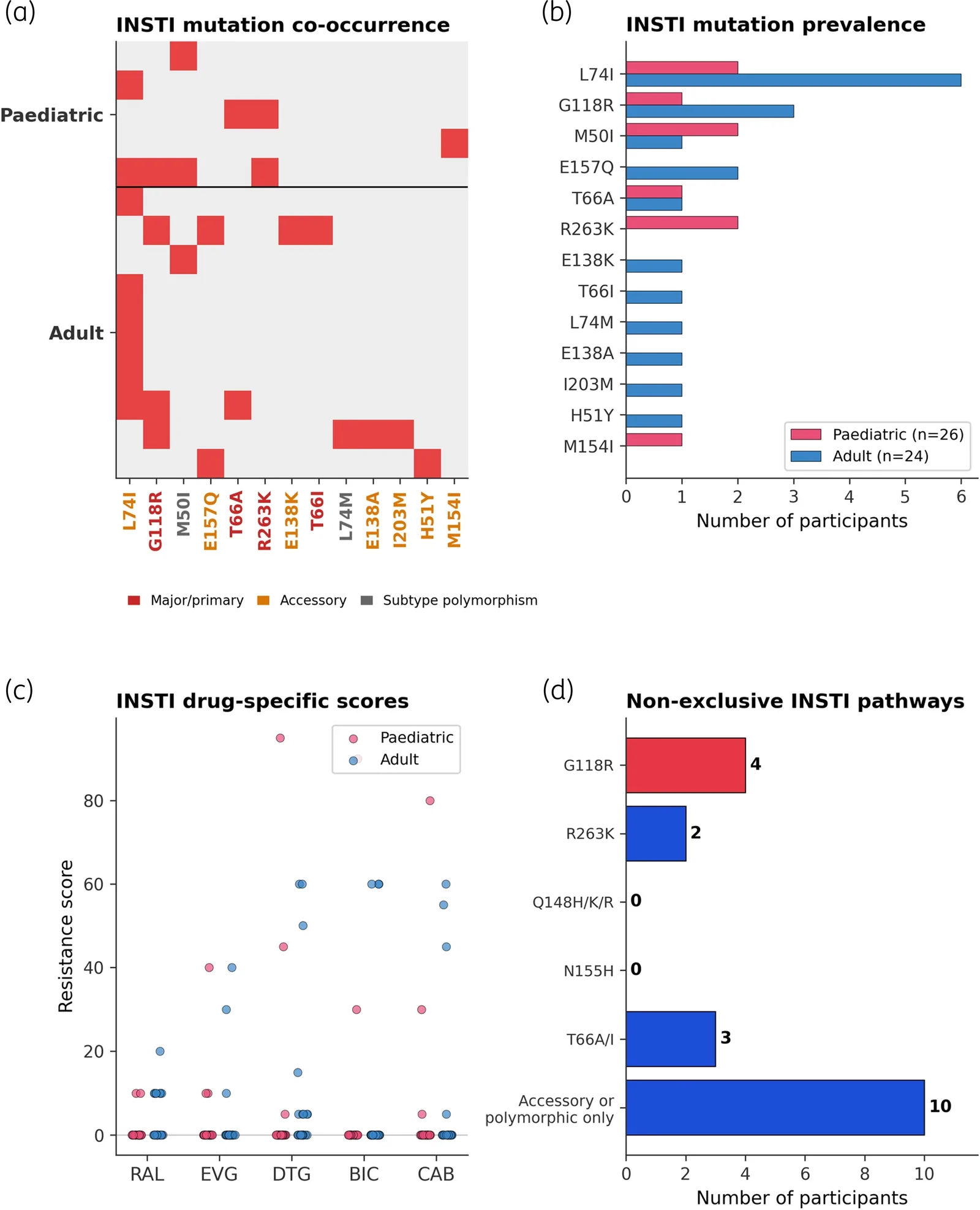
Integrase inhibitor resistance patterns in paediatric and adult cohorts. At custom-catalogue consensus, G118R occurred in four participants and R263K in two; Q148H/K/R and N155H were not detected. (a) Co-occurrence of integrase substitutions. (b) Mutation prevalence by cohort. (c) Drug-specific resistance scores for RAL, EVG, DTG, BIC and CAB. (d) Major observed pathways and participants with accessory or polymorphic substitutions only. L74M is treated as a subtype-associated polymorphism rather than a primary mutation.

**Figure 3.**
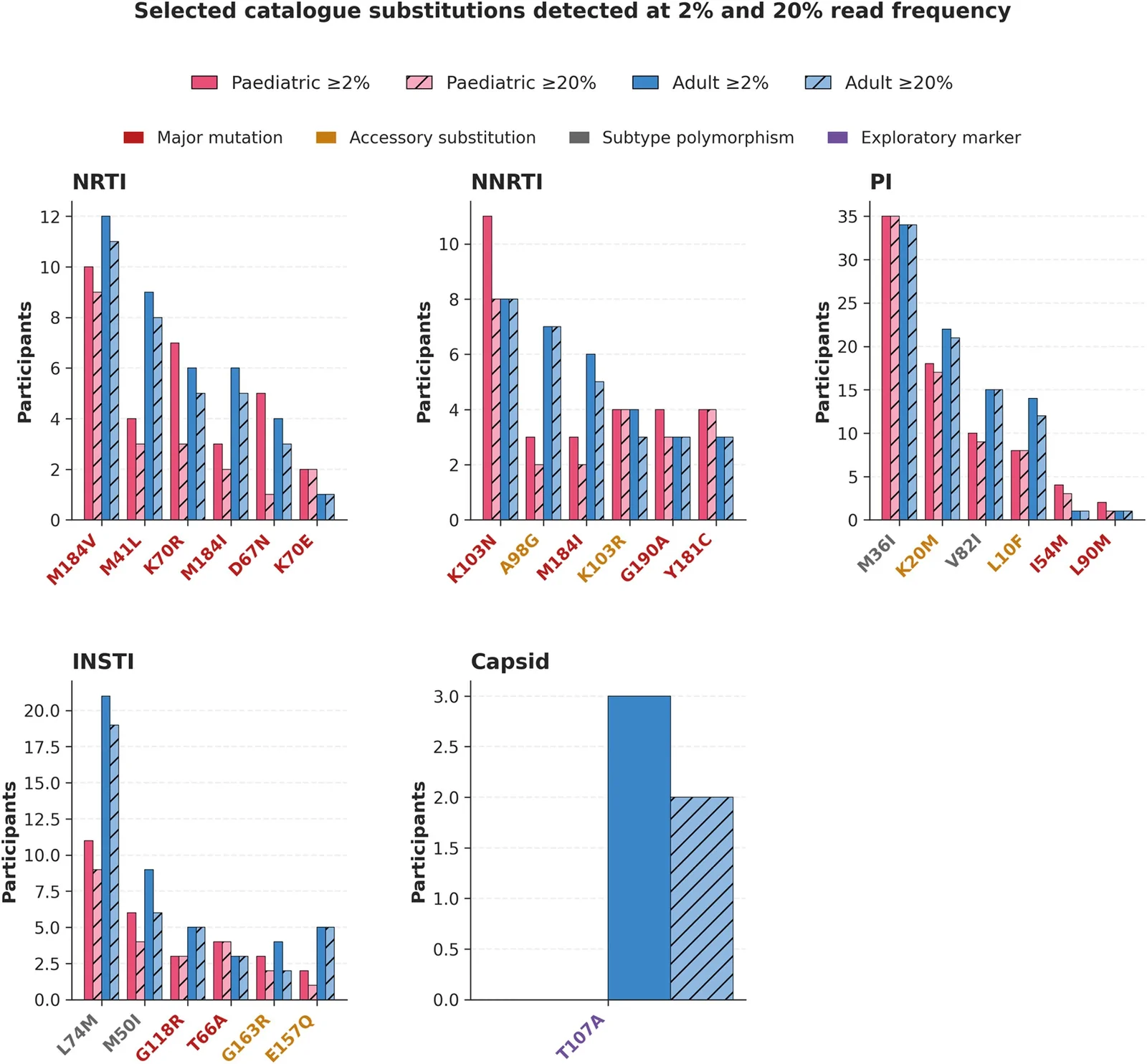
Read-frequency threshold analysis of resistance-associated mutations. Selected catalogue substitutions detected at ≥2% and ≥20% read frequency are shown by age cohort and mutation category, distinguishing major, accessory, subtype-polymorphic and exploratory markers. The analysis is exploratory and does not assign a clinically validated low-frequency threshold.

**Figure 4.**
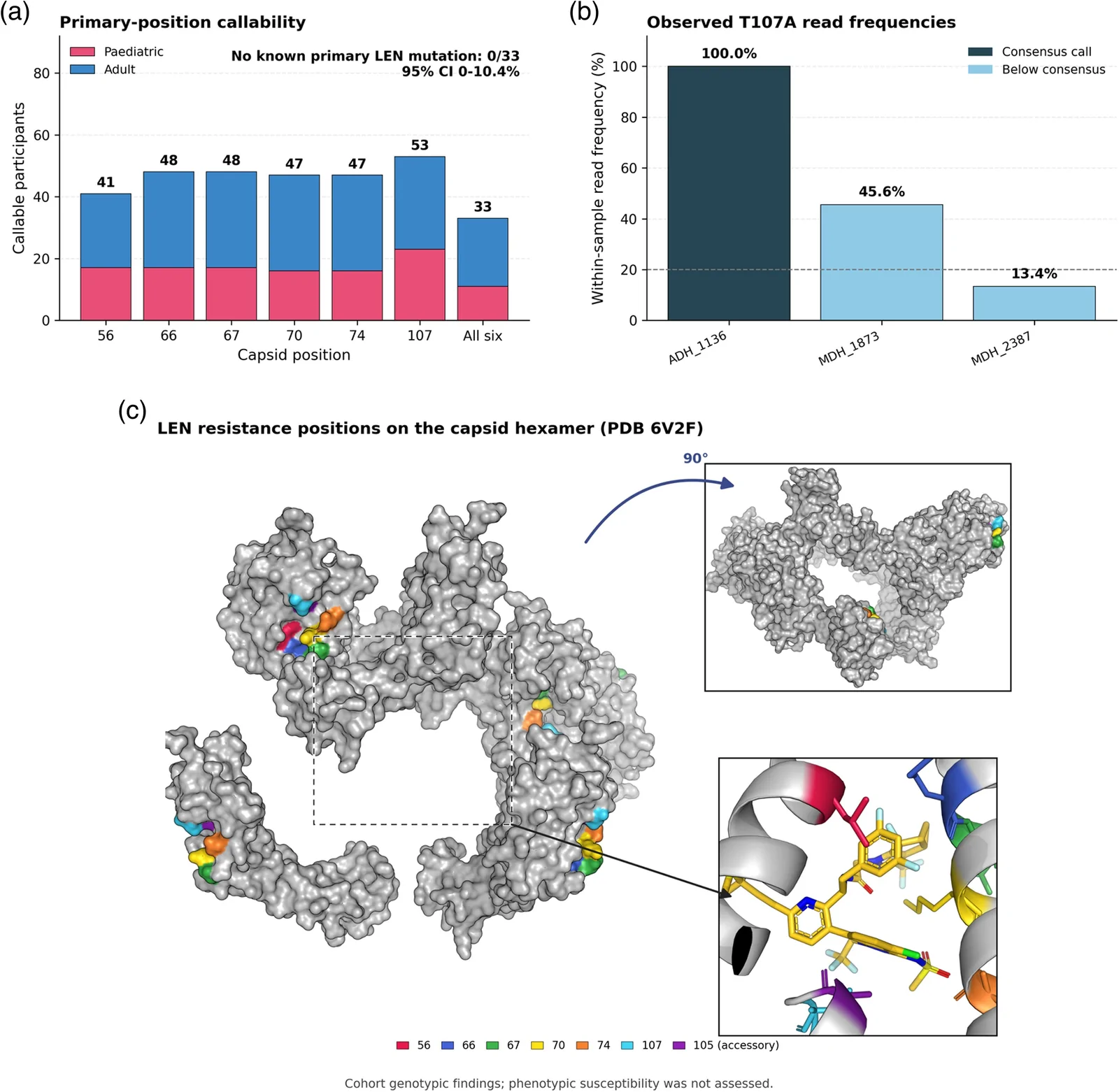
Capsid site callability and LEN-associated variation in the cohort. (a) Callability at the six primary LEN resistance positions; 33 participants had unambiguous sequence at all six positions (22 adults and 11 paediatric participants). No known primary LEN resistance mutation was detected (0/33; 95% CI 0%–10.4%). (b) T107A was detected in three adults: once at consensus and twice below consensus. (c) The six primary LEN resistance positions and accessory position 105 mapped onto the capsid hexamer (PDB 6V2F). These are genotypic findings and do not establish phenotypic susceptibility.

## Data Availability

Raw sequence data will be deposited in the NCBI Sequence Read Archive under BioProject accession PRJNA1505199. Participant-level COMET output is included as Supplementary Data S1. Consensus sequences and other de-identified processed data are available from the corresponding author.

## Code availability

The Snakemake workflow, resistance mutation catalogues and analysis scripts are maintained in a version-controlled repository. The revision-specific code is available from the corresponding author during peer review and will be made publicly available before publication. COMET HIV-1 v2.4 was accessed on 30 July 2026.

## Results

### Study population and sequence characteristics

We analysed 87 HIV-1 samples from participants failing DTG-based ART: *n* = 47 adults (median age 41 years, IQR 35–48) and *n* = 40 paediatric participants (median age 13 years, IQR 9–17). Time since diagnosis for the adult and paediatric cohorts was 12.2 years (IQR 9.1–16.0) and 4.0 years (IQR 1.4–7.9), respectively. These cohort-level summaries used enrolment data; Table 1 reports the subset with linked clinical records. Applying the input-QC rule, 81/87 COMET HIV-1 v2.4 results were assessable for aggregate summaries: subtype G in 18/87, CRF02_AG in 11/87 and A1 in 1/87; 7/87 had other assigned subtype, 44/87 had complex unassigned recombinant profiles and 6/87 were not assessable due to genomic fragmentation (Table S1; Supplementary Data S1). Among the 50 IN-genotypable participants, 9 were subtype G, 6 CRF02_AG, 1 A1, 5 had other or ‘check for’ calls and 29 were unassigned. Gene coverage varied (Figure 1a): protease 74.7% (65/87), reverse transcriptase 47.1% (41/87), integrase 57.5% (50/87) and capsid 46.0% (40/87). Thirty-three participants had callable residues at all six primary LEN resistance positions. APOBEC3G/F hypermutation screening flagged no sequences (0/87).

### Drug resistance across antiretroviral classes

Across the four conventional drug classes, 24/87 participants (27.6%; 95% CI 19.3%–37.8%) had at least one consensus resistance mutation predicted and 9/87 (10.3%; 95% CI 5.5%–18.5%) had resistance in two or more classes (Figure 1a–c). At consensus, NRTI resistance was detected in 8/41 RT-genotypable participants (19.5%; 95% CI 10.2%–34.0%), NNRTI resistance in 10/41 (24.4%; 95% CI 13.8%–39.3%), PI resistance predictions in 9/65 PR-genotypable participants (13.8%; 95% CI 7.5%–24.3%) and predicted INSTI resistance in 13/50 IN-genotypable participants (26.0%; 95% CI 15.9%–39.6%). Seven of the nine PI predictions depended on accessory or subtype-associated substitutions; major PI resistance mutations were present in 2/65 (3.1%; 95% CI 0.8%–10.5%). At the ≥20% read-frequency threshold, predicted suboptimal susceptibility was present in 30/58 NRTI-assessable participants (51.7%; 95% CI 39.2%–64.1%), 32/56 NNRTI-assessable participants (57.1%; 95% CI 44.1%–69.2%) and 34/68 INSTI-assessable participants (50.0%; 95% CI 38.4%–61.6%). At this threshold, M184V was the most common (20 participants), followed by M41L (11), K70R (8), D67N (4), K70E (3), K219Q (2) and Y115F (1) (Figure S1). For NNRTIs, K103N was the most common (16 participants), followed by A98G (9), K103R (7), Y181C (7), G190A (6), V108I (4) and K101E (4) (Figure S2). PI mutation prevalence was largely driven by common non-B polymorphisms, particularly M36I, rather than primary resistance mutations (Figure S3). At consensus, M36I was present in 28/65 PR-genotypable participants, whereas major PI resistance mutations were present in 2/65. Full endpoint definitions and confidence intervals are provided in Table S2.

### INSTI resistance landscape and the G118R pathway

At the ≥20% read-frequency threshold, predicted INSTI suboptimal susceptibility was present in 34/68 assessable participants (50.0%; 95% CI 38.4%–61.6%), and predicted INSTI resistance was present in 13/50 IN-genotypable participants (26.0%; 95% CI 15.9%–39.6%) at consensus. Among 50 individuals with integrase-genotypable virus, 6/50 (12.0%; 95% CI 5.6%–23.8%) had suboptimal susceptibility specifically to DTG: three with very high-level resistance, two with high-level resistance and one with intermediate resistance (Figure 2a and b). Adults contributed 4/24 cases (16.7%; 95% CI 6.7%–35.9%) and paediatric participants 2/26 (7.7%; 95% CI 2.1%–24.1%). The most frequent accessory substitution at consensus was L74I (8/50, 16.0%). L74M was present in 28/67 participants with a callable site at the ≥20% read-frequency threshold and was treated as a common non-B subtype-associated polymorphism rather than a primary INSTI mutation. G118R was detected in 4/50 participants in the custom-catalogue consensus analysis and in 8/47 participants at the ≥20% readback threshold; R263K was present in two participants at consensus (Figure 2c and d). E138K/A was present in 3/50, T66I/A in 3/50, M50I in 3/50 and E157Q in 2/50. None of the IN-genotypable participants carried Q148H/K/R or N155H. COMET classified the four custom-catalogue consensus G118R-positive samples as one CRF02_AG, one subtype G and two unassigned recombinants; the eight participants positive at the ≥20% readback threshold comprised four subtype G, one CRF02_AG and three unassigned recombinants. These small numbers are insufficient to infer subtype-dependent pathway selection. At the 2%, 5%, 10% and 20% thresholds, suboptimal DTG susceptibility was predicted in 20/72, 20/72, 19/72 and 16/72 assessable participants, respectively. These threshold-specific results were exploratory.

### Low-frequency variant detection and resistance scoring

At the ≥2% read-frequency threshold, 443 catalogue substitution calls were detected in 74 participants; 73/87 participants had at least one mutation that contributed to a resistance score. After deduplication by participant, gene and amino acid substitution, 13 calls were present at 2% to <5%, 16 at 5% to <10%, 35 at 10% to <20%, 53 at 20% to <50% and 326 at ≥50%. The polymorphic capsid substitution T107A was detected in three participants: one at consensus and two below consensus. The effect of including low-frequency DRMs in catalogue-based resistance scores was explored (Figure 3). Some participants moved from susceptible to resistant categories when low-frequency INSTI, NRTI and NNRTI mutations were included, although these scores were not treated as validated clinical classifications. No frequency threshold was treated as clinically validated in this cohort (Table S3).

### Paediatric versus adult resistance profiles

The paediatric and adult populations were compared descriptively. Among integrase-genotypable participants, suboptimal DTG susceptibility was detected in 2/26 paediatric participants (7.7%; 95% CI 2.1%–24.1%) and 4/24 adults (16.7%; 95% CI 6.7%–35.9%) (Figure 2a and b; Figure S4). It should be noted that the relatively small numbers and overlapping confidence intervals do not support a firm age-group comparison. Both cohorts showed comparable NRTI mutation profiles, with M184V detected across age groups. No known primary LEN resistance mutation was detected among participants with complete primary-site coverage: 0/11 paediatric participants (95% CI 0%–25.9%) and 0/22 adults (95% CI 0%–14.9%). It should be noted that these findings are based on genotype alone.

### Surveillance drug resistance mutations in treatment-experienced participants

Surveillance mutations were defined using the WHO 2009 SDRM list for NRTI, NNRTI and PI mutations and the Tzou *et al.* (2020) candidate INSTI surveillance mutation list.^13^ At least one consensus SDRM was identified in 13/87 participants (14.9%; 95% CI 8.9%–23.9%). Class-specific prevalence among participants with an evaluable genotype was NRTI 8/41 (19.5%; 95% CI 10.2%–34.0%), NNRTI 10/41 (24.4%; 95% CI 13.8%–39.3%), INSTI 3/50 (6.0%; 95% CI 2.1%–16.2%) and PI 0/65 (0%; 95% CI 0%–5.6%); classes overlap. NNRTI SDRMs included K103N in five participants and Y181C in three. INSTI SDRMs were T66A in two participants and T66I in one. Multi-class SDRMs were identified in six participants. All participants were treatment experienced, and baseline pre-ART genotypes and complete maternal–child histories were unavailable. We therefore report these as SDRMs in treatment-experienced participants and do not infer transmission or apply the WHO pretreatment-resistance 10% policy threshold (Figure S5).

### Baseline capsid variation relevant to LEN resistance

Capsid residues 56, 66, 67, 70, 74 and 107 were callable in 33 participants. No known primary LEN resistance mutation was detected in these samples (0/33; 95% CI 0%–10.4%), comprising 0/22 adults (95% CI 0%–14.9%) and 0/11 paediatric participants (95% CI 0%–25.9%). The polymorphic capsid substitution T107A, whose effect on LEN susceptibility is uncertain, was identified in three participants: at consensus in DOV_ADH_1136 (100% frequency, depth 191×) and below consensus in DOV_MDH_1873 (45.6%, depth 68×) and DOV_MDH_2387 (13.4%, depth 605×). These findings do not establish phenotypic susceptibility (Figure 4; Figure S6).

Comparison with 2847 capsid sequences from the LANL HIV Sequence Database showed that primary LEN resistance substitutions were absent across all subtypes examined (Figure 4b and c) with no difference by cohort. Natural polymorphism at position 105 (A105T) varied by subtype—23.3% in subtype G and 16.7% in CRF02_AG, but A105T alone has not been associated with phenotypic resistance. Position 107 showed limited global variation, with T107N present only in subtype F (3.3%). Taken together, these data show no known primary LEN resistance mutation in the capsid sequences examined. Structural mapping onto the HIV-1 CA hexamer (PDB 6V2F) confirmed that LEN resistance positions cluster within the binding pocket, with residues 66, 67 and 70 making direct contacts with LEN (Figure 4e), whereas position 107 lies adjacent to, but not at, the primary contact interface. These data provide a baseline for surveillance as LEN use expands, but do not establish phenotypic susceptibility or show that pretreatment capsid testing is unnecessary.

### Exploratory analyses of newer agents

These analyses were exploratory genotypic screens and were not used to determine clinical eligibility or efficacy. No primary ENF resistance substitution was detected among 54 assessable participants; this does not establish phenotypic susceptibility to ENF. Coverage was insufficient for participant-level estimates for fostemsavir, ibalizumab and maraviroc, and these agents were not evaluated further. CAB and RPV were both assessable in 34/87 participants: 26/34 were predicted susceptible to both drugs by genotype, 3/34 had low-level RPV resistance and 5/34 had intermediate-or-higher resistance to CAB or RPV. One additional participant had predicted CAB resistance but no evaluable RPV result. Thus, 53/87 lacked a complete paired result. Because all participants had ongoing viraemia and treatment failure, these results do not support immediate switching to CAB + RPV (Table S4; Figure S6).

## Discussion

Understanding how integrase resistance emerges under selective pressure from DTG is important as antiretroviral strategies evolve across sub-Saharan Africa. While LEN targets a distinct viral component,^47,48^ baseline capsid variation remains relevant to future resistance surveillance, although integrase resistance does not imply capsid inhibitor resistance. In settings with expanding DTG use, including much of sub-Saharan Africa,^49–51^ characterizing baseline capsid variation and resistance-associated mutations provides context for future LEN treatment and prevention strategies. Most LEN resistance data derive from heavily treatment-experienced trial participants,^52–54^ with a paucity of data from non-B subtypes that dominate West Africa.

Here, we provide detailed drug resistance profiling from Nigerian adult and paediatric cohorts following the national transition to DTG.^14,55–58^ Across the four conventional drug classes, 24/87 participants had at least one consensus resistance prediction, and 6/50 IN-genotypable participants had suboptimal/compromised DTG susceptibility. Prior NNRTI exposure was recorded in 31/37 adults and 13/36 paediatric participants with linked histories, while 20/36 paediatric participants had previously received ritonavir-boosted lopinavir (LPV/r). The NRTI and NNRTI mutation burden included M184V in 20 participants and K103N in 16.^14,55–58^ G118R was the most frequent major pathway mutation,^59^ occurring in 4/50 participants in the custom consensus analysis and in 8/47 at the ≥20% readback threshold. The four custom consensus cases comprised one CRF02_AG, one subtype G and two unassigned recombinants; these small numbers and the fragmented genomes do not establish subtype-dependent selection.

No known primary LEN resistance mutation was detected in the 33 samples with complete primary-site coverage. Clinical trials reported LEN resistance emergence through Q67H, K70H and N74D,^18,24^ yet we detected none of these. T107A was observed in three participants: one at consensus and two below consensus, but was treated as a polymorphic marker with uncertain susceptibility effect rather than a primary LEN resistance mutation.^60^ The conservation of LEN binding-site residues across CRF02_AG and subtype G is consistent with structural constraints on the capsid hexamer interface^61^ and, combined with the absence of primary LEN substitutions among 2847 global sequences, suggests that known primary LEN resistance substitutions are uncommon before LEN exposure in the sequences examined. This finding is consistent with Marcelin *et al.*,^26^ who found no enrichment of GS-6207 resistance-associated capsid substitutions in protease inhibitor-experienced participants. Neither study included phenotypic testing or outcomes on a LEN-containing regimen.

Detection of score-contributing mutations at ≥2% in 73/87 participants (83.9%) showed that consensus sequencing missed some within-host variants. Pretreatment minority NNRTI resistance predicts 2–4-fold higher failure rates,^62,63^ and minority INSTI mutations associate with virological failure on DTG-based regimens.^62,63^ These low-frequency variants will not be detected by routine consensus sequencing, although their clinical relevance in this cohort is uncertain. We therefore report the 2%, 5%, 10% and 20% threshold analyses separately and do not propose a validated clinical threshold.

Consensus SDRMs were identified in 13/87 participants (14.9%; 95% CI 8.9%–23.9%). NNRTI SDRMs included K103N in five participants and Y181C in three, which may reflect prior NNRTI exposure. INSTI SDRMs were present in three participants (T66A in two and T66I in one). Multi-class SDRMs occurred in six participants. Because all participants were treatment experienced and baseline genotypes were unavailable, these mutations cannot be classified as transmitted resistance.

The resistance profiles were also considered in relation to newer treatment options, but incomplete gene coverage limited these analyses. No primary ENF resistance substitution was detected in 54 assessable participants, although phenotypic susceptibility to ENF was not tested. Among the 34 with paired CAB and RPV results, 26 were predicted susceptible to both, three had low-level RPV resistance and five had intermediate or higher resistance to one or both drugs. These mutation-based predictions were not used to determine suitability for switching treatment. The absence of a known primary capsid resistance mutation does not establish susceptibility or salvage efficacy because LEN phenotypes and treatment outcomes were unavailable; LEN would still require an active optimized background regimen.

DTG suboptimal susceptibility was detected in 2/26 integrase-genotypable paediatric participants (7.7%) and 4/24 adults (16.7%). One paediatric participant (DRESH_WDH_001) harboured G118R, R263K, L74I and M50I. No known primary LEN resistance mutation was detected among participants with complete primary-site coverage in either age group, but this genotypic finding cannot be extrapolated to paediatric efficacy. Detailed maternal ART, PMTCT, prophylaxis, adherence and baseline genotype histories were not uniformly available, so the available histories do not establish when or how this resistance pattern arose.

This study was subject to limitations. The cohort was small, selected for virological failure and not intended to represent the wider Nigerian population. Sampling was cross-sectional, and treatment, adherence and PMTCT histories were incomplete. Gene coverage varied substantially, leaving different denominators for each analysis and the possibility of selection bias. We did not have phenotypic assays or treatment outcomes for LEN or the exploratory newer-agent predictions. Low-frequency calls were not confirmed with technical replicates or unique molecular identifiers, and linkage between variants was not established. Six consensus records lacked a contiguous 100-nt called segment and were treated as not assessable in the aggregate COMET summaries, while many assessable records returned complex or unassigned profiles, limiting subtype-specific inference. Finally, because all participants were treatment experienced, SDRMs observed in this cross-sectional analysis cannot be used to infer transmission. Future work should incorporate long-read sequencing for haplotype reconstruction, longitudinal sampling to track resistance emergence on DTG-based regimens and phenotypic validation of the integrase and capsid findings.

In summary, DTG resistance was detected in both adult and paediatric participants with virological failure, with G118R being the most frequent major mutation. No known primary LEN resistance mutation was detected in the 33 samples with complete primary-site coverage. These findings support continued integrase and capsid surveillance, together with prospective phenotypic and clinical evaluation of LEN-containing regimens.

## Supplementary data

Figures S1–S6 and Tables S1–S4 are available as Supplementary data at JAC Online.
