# Supplementary material for: HIV-1 integrase inhibitor resistance in viraemic-treated individuals and baseline capsid diversity relevant to future lenacapavir therapy
Source: J Antimicrob Chemother. 2026 Sep 8;81(10):dkag300. doi: 10.1093/jac/dkag300 (PMC13551618; doi:10.1093/jac/dkag300)
Supplement: Supplementary file 1 [file dkag300_supplementary_data.docx]

**Supplementary material**

# Supplementary Methods

COMET subtyping. All participant consensus FASTA records were submitted in one batch to COMET HIV-1 v2.4 on 30 July 2026. The service-reported subtype or recombinant label and bootstrap support were retained where available. Records were required to contain at least one contiguous 100-nt unambiguous segment; six records failing this local input-QC check were treated as not assessable in aggregate summaries irrespective of the returned service string. No BLAST reassignment was applied. Complex COMET outputs were grouped only for aggregate summaries, and the exact returned string is provided in Supplementary Data S1.

Low-frequency quality control. Calls required two alternate codon-supporting read alignments, bidirectional support, mapping quality >=20, base quality >=15 at every codon position, and sufficient threshold-specific codon depth. Overlapping mates were not collapsed to unique molecules. No negative controls, empirical sequencing/PCR error estimate, technical replicates or unique molecular identifiers were available, so these analyses are exploratory.

**Supplementary Table S1. Strict-QC COMET HIV-1 classifications in the full cohort and integrase analysis subsets.**

| Population | N | G | CRF02_AG | A1 | Other assigned/check | Unassigned recombinant | Not assessable |
| --- | --- | --- | --- | --- | --- | --- | --- |
| All participants | 87 | 18/87 (20.7) | 11/87 (12.6) | 1/87 (1.1) | 7/87 (8.0) | 44/87 (50.6) | 6/87 (6.9) |
| IN-genotypable | 50 | 9/50 (18.0) | 6/50 (12.0) | 1/50 (2.0) | 5/50 (10.0) | 29/50 (58.0) | 0/50 (0) |
| G118R-callable at readback | 47 | 12/47 (25.5) | 9/47 (19.1) | 0/47 (0) | 2/47 (4.3) | 24/47 (51.1) | 0/47 (0) |
| Custom-catalogue consensus G118R-positive | 4 | 1/4 (25.0) | 1/4 (25.0) | 0/4 (0) | 0/4 (0) | 2/4 (50.0) | 0/4 (0) |
| Stanford HIVdb consensus G118R-positive | 7 | 4/7 (57.1) | 1/7 (14.3) | 0/7 (0) | 0/7 (0) | 2/7 (28.6) | 0/7 (0) |
| >=20% readback G118R-positive | 8 | 4/8 (50.0) | 1/8 (12.5) | 0/8 (0) | 0/8 (0) | 3/8 (37.5) | 0/8 (0) |

**Supplementary Table S2. Consensus and exploratory read-frequency resistance estimates with endpoint-specific denominators.**

| Analysis | Endpoint | n/N | % | Wilson 95% CI | Denominator definition |
| --- | --- | --- | --- | --- | --- |
| Consensus | Any predicted resistance across NRTI/NNRTI/PI/INSTI custom scores | 24/87 | 27.6 | 19.3-37.8 | Full cohort; any positive custom score retained across evaluable conventional classes |
| Consensus | Resistance in >=2 conventional classes | 9/87 | 10.3 | 5.5-18.5 | Full cohort; positive custom scores in >=2 conventional classes |
| Consensus | NRTI resistance | 8/41 | 19.5 | 10.2-34.0 | RT-genotypable participants |
| Consensus | NNRTI resistance | 10/41 | 24.4 | 13.8-39.3 | RT-genotypable participants |
| Consensus | Major PI DRM | 2/65 | 3.1 | 0.8-10.5 | PR-genotypable participants |
| Consensus | Predicted INSTI resistance | 13/50 | 26.0 | 15.9-39.6 | IN-genotypable participants |
| Consensus | Major INSTI DRM | 5/50 | 10.0 | 4.3-21.4 | IN-genotypable participants |
| Consensus | DTG non-susceptibility | 6/50 | 12.0 | 5.6-23.8 | IN-genotypable participants |
| Consensus | Adult DTG non-susceptibility | 4/24 | 16.7 | 6.7-35.9 | Adult IN-genotypable participants |
| Consensus | Paediatric DTG non-susceptibility | 2/26 | 7.7 | 2.1-24.1 | Paediatric IN-genotypable participants |
| Consensus | Custom-catalogue G118R | 4/50 | 8.0 | 3.2-18.8 | IN-genotypable participants in the custom consensus scorer |
| Consensus | SDRM in treatment-experienced participants | 13/87 | 14.9 | 8.9-23.9 | Full treatment-experienced cohort |
| Consensus SDRM | NRTI SDRM | 8/41 | 19.5 | 10.2-34.0 | RT-genotypable participants |
| Consensus SDRM | NNRTI SDRM | 10/41 | 24.4 | 13.8-39.3 | RT-genotypable participants |
| Consensus SDRM | PI SDRM | 0/65 | 0 | 0-5.6 | PR-genotypable participants |
| Consensus SDRM | INSTI SDRM | 3/50 | 6.0 | 2.1-16.2 | IN-genotypable participants |
| Consensus | Primary LEN DRM | 0/33 | 0 | 0-10.4 | Complete callability at CA residues 56, 66, 67, 70, 74 and 107 |
| Consensus | Adult primary LEN DRM | 0/22 | 0 | 0-14.9 | Adult participants with complete callability at all six primary LEN positions |
| Consensus | Paediatric primary LEN DRM | 0/11 | 0 | 0-25.9 | Paediatric participants with complete callability at all six primary LEN positions |
| >=20% reads | Predicted NRTI non-susceptibility | 30/58 | 51.7 | 39.2-64.1 | NRTI read-frequency class panel assessable at >=20% |
| >=20% reads | Predicted NNRTI non-susceptibility | 32/56 | 57.1 | 44.1-69.2 | NNRTI read-frequency class panel assessable at >=20% |
| >=20% reads | Predicted INSTI non-susceptibility | 34/68 | 50.0 | 38.4-61.6 | INSTI read-frequency class panel assessable at >=20% |
| >=20% reads | Readback G118R | 8/47 | 17.0 | 8.9-30.1 | Callable IN codon 118 at >=20% read frequency |
| >=2% reads | Predicted DTG non-susceptibility | 20/72 | 27.8 | 18.8-39.0 | DTG read-frequency panel assessable at >=2% |
| >=5% reads | Predicted DTG non-susceptibility | 20/72 | 27.8 | 18.8-39.0 | DTG read-frequency panel assessable at >=5% |
| >=10% reads | Predicted DTG non-susceptibility | 19/72 | 26.4 | 17.6-37.6 | DTG read-frequency panel assessable at >=10% |
| >=20% reads | Predicted DTG non-susceptibility | 16/72 | 22.2 | 14.2-33.1 | DTG read-frequency panel assessable at >=20% |

**Supplementary Table S3. Low-frequency variant quality control, callability and observed catalogue substitutions.**

| Metric | Value |
| --- | --- |
| DRM site-participant pairs | 7,482: 87 participants x 86 sites |
| Sites by gene | CA 7; IN 21; PR 25; RT 33 |
| Minimum codon depth, median (IQR) | 321 (0-1,391) |
| >=100 reads | 4,592/7,482 (61.4%) |
| >=40 reads | 4,876/7,482 (65.2%) |
| >=20 reads | 5,002/7,482 (66.9%) |
| >=10 reads | 5,081/7,482 (67.9%) |
| Threshold/minimum depth | 2%/100; 5%/40; 10%/20; 20%/10 |
| Alternate-read rule | >=2 codon-supporting read alignments, including >=1 per strand; overlapping mates were not collapsed to unique molecules |
| Read/base filters | Mapping quality >=20; all three codon bases Q15; duplicate, secondary, supplementary, QC-failed and unmapped reads excluded |
| Strand-bias test | No formal P-value applied |
| Empirical error control | No negative control or empirical sequencing/PCR error estimate available |
| Panel callability | Calculated per drug; a negative call required >=30% callable panel positions; detected evidence was retained |
| Technical replicates/UMIs | Not available |
| Deduplicated participant-gene-substitution calls | 443 in 74 participants |
| Participants with score-contributing mutation | 73/87 |
| Frequency bins | 13 at 2%-<5%; 16 at 5%-<10%; 35 at 10%-<20%; 53 at 20%-<50%; 326 at >=50% |

**Supplementary Table S4. Sequence evaluability for exploratory ENF and CAB/RPV analyses.**

| Analysis | Evaluable definition/result | n/N |
| --- | --- | --- |
| ENF | Complete primary HR1 panel; non-polymorphic primary substitution detected | 0/54 |
| CAB | Consensus prediction evaluable | 50/87 |
| RPV | Consensus prediction evaluable | 41/87 |
| CAB+RPV | Both predictions evaluable | 34/87 |
| CAB+RPV paired | Susceptible to both | 26/34 |
| CAB+RPV paired | CAB susceptible with low-level RPV resistance | 3/34 |
| CAB+RPV paired | Intermediate-or-higher resistance to either drug | 5/34 |
| Unpaired | CAB resistance with RPV not evaluable | 1/87 |
| Other incomplete | Remaining incomplete paired assessments | 52/87 |

Supplementary Table S1 contains the exact participant-level COMET output, input sequence support, strict aggregate category, IN callability and G118 readback callability at the >=20% threshold, and separate custom-catalogue, Stanford HIVdb consensus and >=20% readback G118R indicators. Per-input SHA-256 values are also included.

Supplementary Figure 1: NRTI-associated mutation prevalence. At the >=20% read-frequency threshold, M184V was most prevalent (20 participants), followed by M41L (11), K70R (8), D67N (4), K70E (3), K219Q (2), and Y115F (1). Consensus counts are shown separately.
Alt text: Grouped bars compare consensus and 20% threshold counts for NRTI-associated substitutions.

Supplementary Figure 2: NNRTI-associated mutation prevalence. At the >=20% read-frequency threshold, K103N was most prevalent (16 participants), followed by A98G (9), K103R (7), Y181C (7), G190A (6), V108I (4), and K101E (4). Consensus counts are shown separately.
Alt text: Grouped bars compare consensus and 20% threshold counts for NNRTI-associated substitutions.

Supplementary Figure 3: PI-associated mutation prevalence, separating subtype-associated polymorphisms from established resistance mutations. M36I, K20I, V82I, and T74S are shown as polymorphisms and are not interpreted as primary PI resistance.
Alt text: Bar chart separates common non-B protease polymorphisms from less frequent established PI resistance mutations

Supplementary Figure 4: Exploratory threshold-specific predicted DTG non-susceptibility. Among 72 participants assessable at each threshold, predicted non-susceptibility was detected in 16/72 at 20%, 19/72 at 10%, 20/72 at 5% and 20/72 at 2% read frequency. Adult and paediatric counts are shown separately. No threshold is presented as clinically validated.
Alt text: Grouped bars show adult and paediatric predicted DTG non-susceptibility at 2%, 5%, 10% and 20% read-frequency thresholds.

Supplementary Figure 5: Surveillance drug resistance mutations in treatment-experienced participants. Class-specific prevalence uses gene-genotypable denominators: NRTI 8/41, NNRTI 10/41, PI 0/65 and INSTI 3/50. These findings are not classified as transmitted resistance.
Alt text: Four bars show the percentage of gene-genotypable participants with consensus SDRMs in each drug class.

Supplementary Figure 6: Participant-level callability at the six primary LEN resistance positions. Thirty-three participants had unambiguous sequence at all six positions, comprising 22 adults and 11 paediatric participants. A negative primary-resistance result was assigned only to these complete-panel participants.
Alt text: A participant-by-position matrix shows callability at capsid residues 56, 66, 67, 70, 74 and 107, with complete coverage in 33 participants.
